# Supplementary material for: Vitamin D Modified DSS-Induced Colitis in Mice via STING Signaling Pathway
Source: Biology (Basel). 2025 Jun 18;14(6):715. doi: 10.3390/biology14060715 (PMC12190092; doi:10.3390/biology14060715)
Supplement: Supplementary file 1 [file biology-14-00715-s001.zip › Supplementary Materials S1: 16s-report of gut microbiota/Groups/diff_analysis/RF/Genus_Groups.RFimportance.pdf]

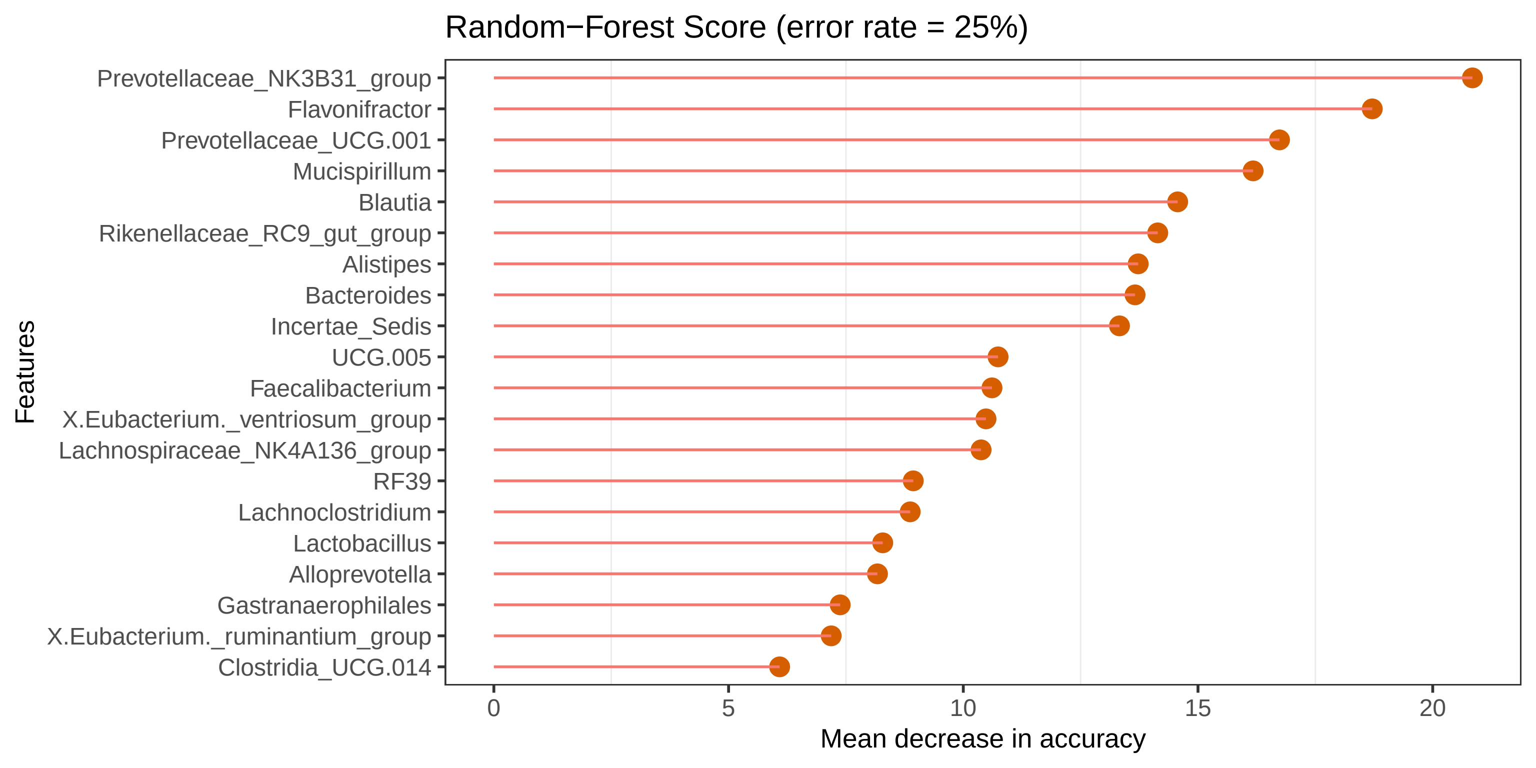

Random−Forest Score (error rate = 25%)
Prevotellaceae_NK3B31_group
Flavonifractor
Prevotellaceae_UCG.001
Mucispirillum
Blautia
Rikenellaceae_RC9_gut_group
Alistipes
Bacteroides
Incertae_Sedis
UCG.005
Faecalibacterium
X.Eubacterium._ventriosum_group
Lachnospiraceae_NK4A136_group
RF39
Lachnoclostridium
Lactobacillus
Alloprevotella
Gastranaerophilales
X.Eubacterium._ruminantium_group
Clostridia_UCG.014
Features
0
5
10
15
20
Mean decrease in accuracy
